# POD-1/*Tcf21* overexpression reduces endogenous SF-1 and StAR expression in rat adrenal cells

**DOI:** 10.1590/1414-431X20154748

**Published:** 2015-09-29

**Authors:** M. M. França, N. P. Abreu, T. A. M. Vrechi, C. F. Lotfi

**Affiliations:** Departamento de Anatomia, Instituto de Ciências Biomédicas, Universidade de São Paulo, São Paulo, SP, Brasil

**Keywords:** POD-1, Tcf21, SF-1, Rat adrenal cell primary culture, Glomerulosa cells, Fasciculata cells

## Abstract

During gonad and adrenal development, the POD-1/capsulin/*TCF21*transcription factor negatively regulates *SF-1/NR5A1*expression, with higher SF-1 levels being associated with increased adrenal cell proliferation and tumorigenesis. In adrenocortical tumor cells, POD-1 binds to the *SF-1* E-box promoter region, decreasing *SF-1* expression. However, the modulation of SF-1 expression by POD-1 has not previously been described in normal adrenal cells. Here, we analyzed the basal expression of *Pod-1* and *Sf-1* in primary cultures of glomerulosa (G) and fasciculata/reticularis (F/R) cells isolated from male Sprague-Dawley rats, and investigated whether POD-1 overexpression modulates the expression of endogenous *Sf-1* and its target genes in these cells. POD-1 overexpression, following the transfection of pCMVMycPod-1, significantly decreased the endogenous levels of *Sf-1* mRNA and protein in F/R cells, but not in G cells, and also decreased the expression of the SF-1 target StAR in F/R cells. In G cells overexpressing POD-1, no modulation of the expression of SF-1 targets, StAR and CYP11B2, was observed. Our data showing that G and F/R cells respond differently to ectopic POD-1 expression emphasize the functional differences between the outer and inner zones of the adrenal cortex, and support the hypothesis that SF-1 is regulated by POD-1/*Tcf21* in normal adrenocortical cells lacking the alterations in cellular physiology found in tumor cells.

## Introduction

The adrenal cortex contains a capsule that surrounds three functionally distinct zones that are arranged around the medulla: the zona glomerulosa, immediately beneath the capsule, which secretes mineralocorticoids, and the inner zona fasciculata and zona reticularis, which secrete glucocorticoids and androgens, respectively. The adrenal cortex is composed of cells specialized in the production of steroids that control mammalian homeostasis.

The splicing factor 1 (SF-1, encoded by *NR5A1*) transcription factor is a key regulator of the expression of steroidogenic enzymes, and is essential for the production of steroid hormones in adrenocortical cells (1,2). Moreover, SF-1 is essential for adrenal development (3,4). An excess of SF-1 induces the proliferation of peripheral adrenocortical cells (5), whereas SF-1 deficiency prevents growth of the contralateral adrenal gland and gonads after unilateral adrenalectomy in mice, and causes adrenal disorders in humans (6,7).

Protein-DNA binding assays and transfection experiments have demonstrated that the basal *Sf-1* promoter interacts with transcription factors such as the CCAAT-box binding factor, stimulatory proteins 1 and 3, and the Polycomb M33 factor (8-11). Furthermore, the helix-loop-helix upstream stimulatory factors 1 and 2 (USF1/2) increase *Sf-1* transcription through interactions with an E-box binding site spanning the −81/−76 region of the *Sf-1*promoter (9,12-14). POD-1/capsulin/*Tcf21* is another helix-loop-helix factor implicated in transcriptional regulation after binding to the E-box element of the *Sf-1* promoter. However, in contrast to USF1/2, POD-1 represses the expression of *Sf-1* in fetal mouse testis by preventing the binding of USF1/2 to the *Sf-1* E-box sequence (13,15). Deletion of *Pod-1* leads to increased expression of SF-1 during testis development, and results in the premature commitment of progenitor cells to the steroidogenic lineage because of the expression of *Sf-1* target genes (13). Thus, there is strong evidence to suggest that POD-1/*Tcf21* is involved in the regulation of SF-1 expression in the testis; however, it is unclear whether POD-1/*Tcf21* plays a role in the control of SF-1 expression in the adrenal cortex.

Recently, we reported that in human adrenocortical tumor cells, exogenous POD-1 binds to the *SF-1* E-box sequence and inhibits SF-1 expression as well as the expression of the steroidogenic acute regulatory protein (StAR) (16), which is responsible for the initial hormone-dependent and rate-limiting step of cholesterol transport during steroidogenesis (17). However, tumor cells have an abnormal physiology, so it has remained unclear whether POD-1 also controls SF-1 expression in normal, non-transformed adrenal cells. In the present study, we examined POD-1 and SF-1 expression in primary cultures of isolated rat adrenal cells, specifically glomerulosa (G) and fasciculata/reticularis (F/R) cells, that were transiently transfected with a vector for POD-1 overexpression. In F/R cells, but not in G cells, POD-1 overexpression inhibited endogenous *Sf-1*/SF-1 expression. Our results therefore support the hypothesis that SF-1 is regulated by POD-1/*Tcf21* in normal primary adrenocortical cells.

## Material and Methods

### Preparation of primary adrenal cell cultures

Adult male Sprague-Dawley rats (body weight: 250-300 g; 7-10 rats per culture) were obtained from the animal facility of the Instituto de Ciências Biomédicas (Universidade de São Paulo, São Paulo, SP, Brasil). The Ethics Care Committee approved the experimental protocol (#83/10). Animals were maintained on a 12-h light/dark cycle and at a controlled temperature, with food and water available *ad libitum*. Animals were sacrificed between 07:00 and 09:00 h by decapitation, and their adrenal glands were promptly removed.

Briefly, G and F/R cells were obtained by sequential collagenase digestion and the mechanical disaggregation of rat adrenal tissue, as previously described and characterized (18,19). Cells were resuspended in DMEM (Gibco, USA) containing 10% FBS (Gibco, Brazil), 25 mg/L ampicillin, and 100 mg/L streptomycin (Sigma Aldrich Gmbh, Germany), and then seeded onto primary tissue culture dishes (Becton Dickinson Labware, USA). Before use, the cells were cultured for 24 h at 37°C in a humidified atmosphere of 5% CO_2_.

### Transfection assay

G and F/R cells were transiently transfected with pCMVMycPod-1 (kindly provided by Dr. Masataka Nakamura, Tokyo Medical University, Japan) as previously described (20). Briefly, 2 × 10^5^ or 5 × 10^5^ cells (for RNA or protein extraction, respectively) were plated onto six-well plates or 60 mm primary tissue culture dishes (Becton Dickinson Labware) and transfected with plasmid DNA using the X-tremeGENE HP DNA transfection reagent¯ (Roche Diagnostics GmbH, Germany), in the proportion of 1 µg plasmid DNA and 1 µL transfection reagent per 1 × 10^5^ cells.

### Total RNA extraction and quantitative reverse transcription PCR (qRT-PCR)

For qRT-PCR analysis, total RNA was extracted from lung and testicular tissues after tissue maceration in a homogenizer, and from primary rat adrenal cells (untransfected or 48 h post-transfection) using the TRIzol^¯^reagent (Invitrogen, USA). Total RNA was treated with TURBO DNA-free™ DNase (Ambion Inc., USA) before cDNA production from 1 µg total RNA using an 1 µL oligo(dT) primer (500 µg/ml), 1 µL RNase-OUT (40 units/µL), and 1 µL M-MLV reverse transcriptase (Invitrogen) according to the manufacturer's instructions. qRT-PCR was performed in a Corbett Rotor-Gene 6000 sequence detector (Qiagen, USA) using the Platinum SYBR qPCR SuperMix-UDG (Invitrogen). The following primers were used: *Sf-1* forward: 5′-GCTGTGTGTTTGGGATGATG-3′ and reverse: 5′-AGACGGAGGAAGGAGTGGTT-3′; *Pod-1* forward: 5′-GCTCTCCAAGCTGGACACTC-3′ and reverse: 5′-ACACCTCCAAGGTCAGGATG-3′; *Star* forward: 5′-TCAGAGTAGCAGCTCCCTTGTTTG-3′ and reverse: 5′-CTCCAAATCCTGAAACGGGAATGC-3′. A cycle threshold (Ct) value in the log range of amplification was selected for each sample in triplicate and was normalized to β-actin expression levels. Reactions were carried out in triplicate. Data were analyzed using the 2^−ΔΔCt^method (21).

### Sodium dodecyl sulfate polyacrylamide gel electrophoresis (SDS-PAGE) and immunoblotting

For Western blotting, cells were lysed 72 h post-transfection in lysis buffer, containing protease and phosphatase inhibitors (Sigma Aldrich Gmbh). The insoluble fraction was removed by centrifugation at 16,000 *g*for 15 min at 4°C, and the protein concentration in supernatants was determined using the Bradford assay. Total protein lysates (30 µg) were resolved by 12% SDS-PAGE and, after electrophoresis, gels were blotted onto nitrocellulose membranes. Non-specific binding sites were blocked for 1.5 h at room temperature with 5% non-fat dried milk in Tris buffered saline solution containing 1% Tween 20 (TBST). All washes and antibody incubations were performed using TBST. The following primary antibodies were used (at dilution ratios of 1:1000): anti-SF-1 (Abcam; ab79377), anti-StAR (ab58013) or anti-CYP11B2 (ab167413), and anti-α-actinin (Santa Cruz; sc-15355). Proteins were visualized by ECL detection with secondary horseradish peroxidase-conjugated anti-rabbit (Amersham Hybond ECL, Germany) or anti-mouse antibodies (Jackson Immuno Research, USA). Immunoblot results were quantified by densitometry using GeneSnap and GeneTools software (SynGene-Synoptic Ltd., UK). Ponceau staining of the membranes was used to monitor protein transfer and loading.

### Statistical analysis

Data are reported as means±SD of three replicate experiments. Each PCR reaction was carried out in triplicate. Statistical significance was determined using paired *t*-tests or one-way analysis of variance (ANOVA) as indicated in the Figure legends. The results were considered statistically significant when P<0.05.

## Results

### *Sf-1* expression is higher in F/R cells than in G cells

**Figure 1:**
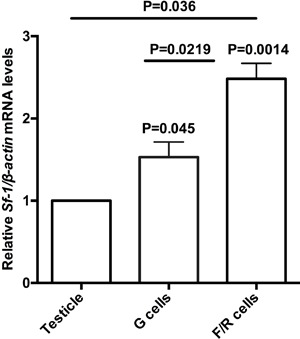
Quantitative reverse transcription PCR (qRT-PCR) analysis of relative *Sf-1/β-actin* mRNA levels in testicular tissue, used as a normalizer, and in rat adrenal glomerulosa (G) and fasciculata/reticularis (F/R) primary cells. Data are reported as the means±SD from three experiments. Statistical significance was assessed by paired *t*-tests and one-way ANOVA.

**Figure 2:**
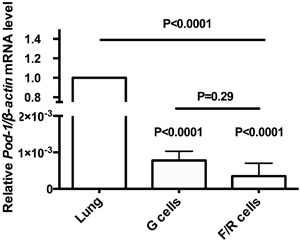
Quantitative reverse transcription RT-PCR (qRT-PCR) analysis of relative *Pod-1/β-actin* mRNA levels in lung tissue, used as a normalizer, and in rat adrenal glomerulosa (G) and fasciculata/reticularis (F/R) primary cells. Data are reported as the means±SD from three experiments. Statistical significance was assessed by paired *t*-tests and one-way ANOVA.

We used qRT-PCR to estimate the endogenous mRNA levels of *Sf-1*and *Pod-1* in isolated rat G and F/R primary cells of the adrenal cortex. *Sf-1* mRNA levels were significantly higher in adrenal cells by 1.53±0.31 (P=0.045) and 2.48±0.32 (P=0.0014) fold in G and F/R cells, respectively, than in testicular tissue, which also expresses high levels of SF-1 (Figure 1). Moreover, *Sf-1* expression was significantly higher in F/R cells (0.95± 0.26 fold; P=0.0219) than in G cells. By contrast, *Pod-1* mRNA was barely detectable in G and F/R cells when compared with lung tissue (Figure 2), where *Pod-1* is expressed in normal epithelial cells and is abnormally methylated and silenced in lung cancers (22). Furthermore, *Pod-1* mRNA levels were not significantly different (P=0.29) between G and F/R cells.

### POD-1 overexpression reduces the endogenous levels of *Sf-1*/SF-1 mRNA and protein in F/R cells

**Figure 3:**
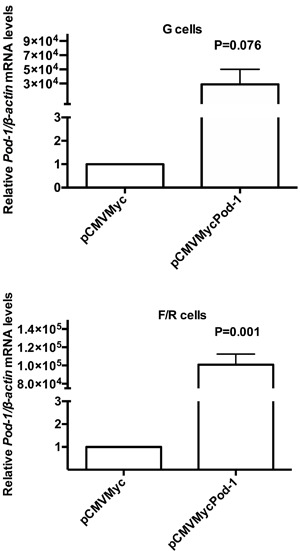
Quantitative reverse transcription RT-PCR (qRT-PCR) analysis of relative *Pod-1/β-actin* mRNA levels in primary rat adrenal glomerulosa cells (G cells) and fasciculata/reticularis cells (F/R cells) that had been transiently transfected with the empty vector pCMVMyc or with pCMVMycPod-1. Total RNA samples used in qRT-PCR were prepared 48 h post-transfection. Data are reported as the means±SD from three experiments. Statistical significance was assessed by paired *t*-tests.

The transient transfection of pCMVMycPod-1 in G and F/R cells resulted in increased *Pod-1* expression in both cell types, compared with controls transfected with empty vector (pCMVMyc) (Figure 3). In mRNA samples prepared 48 h post-transfection of pCMVMycPod-1, 2.89 × 10^4^ (P=0.076) and 1.02 × 10^5^(P=0.001) fold increases in the expression of *Pod-1* were observed in G and F/R cells, respectively (Figure 3).

**Figure 4:**
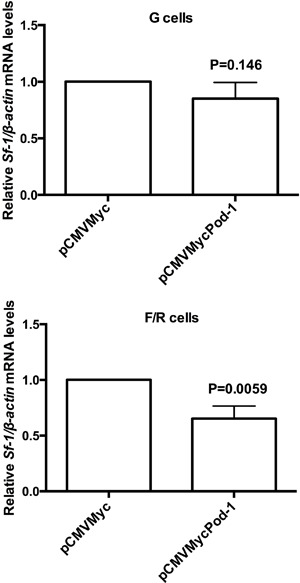
Quantitative reverse transcription PCR (qRT-PCR) analysis of relative *Sf-1/ β-actin* mRNA levels in primary rat adrenal glomerulosa cells (G cells) and fasciculata/reticularis cells (F/R cells) that had been transiently transfected with the empty vector pCMVMyc or with pCMVMycPod-1. Total RNA samples used in qRT-PCR were prepared 48 h post-transfection. Data are reported as the means±SD of three experiments. Statistical significance was assessed by paired *t*-tests.

**Figure 5:**
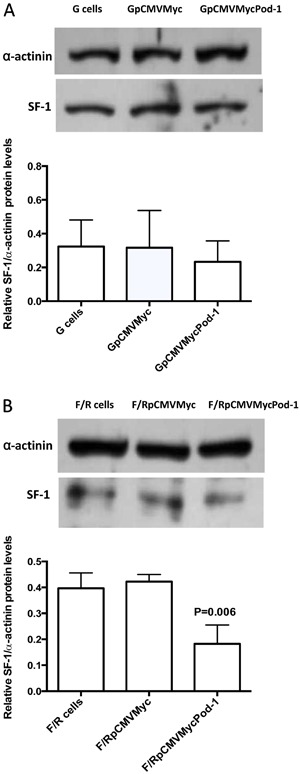
Immunoblotting analysis of relative SF-1/α-actinin protein levels in primary rat adrenal glomerulosa cells (G cells; *A*) and fasciculata/reticularis cells (F/R cells; *B*) untransfected or transiently transfected with the empty vector pCMVMyc or with pCMVMycPod-1. Protein samples were prepared 72 h post-transfection. Data are reported as the means±SD from three experiments. Statistical significance was assessed by paired *t*-tests between cells transfected with pCMVMycPod-1 or empty vector.

To investigate whether POD-1 overexpression affects *Sf-1*/SF-1 mRNA and protein levels, we performed qRT-PCR and immunoblotting analysis of G and F/R cells that had been transiently transfected with the expression vector pCMVMycPod-1 (Figures 4 and 5). In G cells, the transfection of pCMVMycPod-1 did not significantly modulate *Sf-1/*SF-1 mRNA (P=0.146) or protein (P=0.75) levels compared with transfected controls (pCMVMyc) (Figures 4 and 5, respectively). However, by contrast in F/R cells, *Sf-1/*SF-1 mRNA and protein levels were significantly reduced by 0.35- (P=0.0059) and 0.24-fold (P=0.006), respectively, compared with transfected controls (Figures 4 and 5, respectively).

### Decreased SF-1 expression, induced by POD-1 overexpression, reduces StAR protein levels in F/R cells

**Figure 6:**
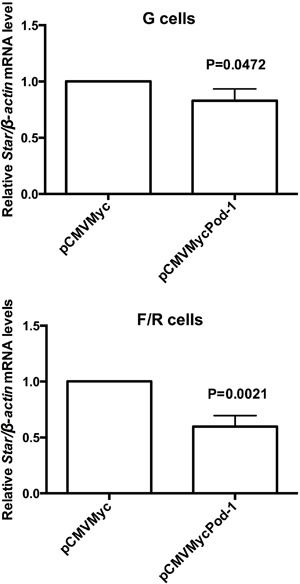
Quantitative reverse transcription PCR (qRT-PCR) analysis of relative *Star/β-actin* mRNA levels in primary rat adrenal glomerulosa cells (G cells) and fasciculata/reticularis cells (F/R cells) that had been transiently transfected with the pCMVMyc (empty vector) or pCMVMycPod-1. mRNA samples were prepared 48 h post-transfection. Data are reported as the means±SD from three experiments. Statistical significance was assessed by paired *t*-tests.

**Figure 7:**
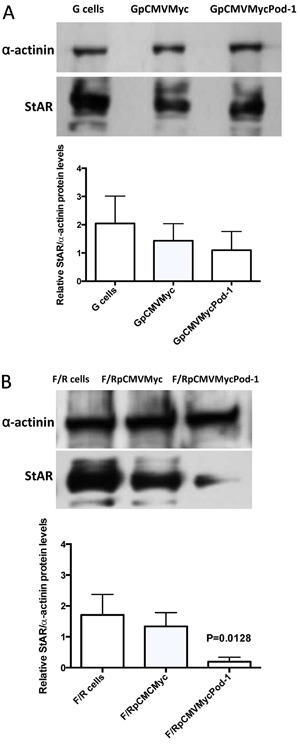
Immunoblotting analysis of relative StAR/α-actinin protein levels in primary rat adrenal glomerulosa cells (G cells; *A*) and fasciculata/reticularis cells (F/R cells; *B*) untransfected or transiently transfected with the empty vector pCMVMyc or with pCMVMycPod-1. Protein samples were prepared 72 h post-transfection. Data are reported as the means±SD from three experiments. Statistical significance was assessed by paired *t*-tests between cells transfected with pCMVMycPod-1 or empty vector.

**Figure 8:**
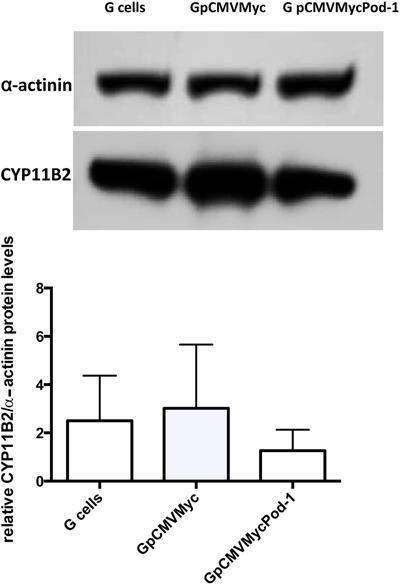
Immunoblotting analyses of relative CYP11B2/α-actinin protein levels in primary adrenal glomerulosa cells (G cells), untransfected G cells, and G cells transiently transfected with the empty vector pCMVMyc or with pCMVMycPod-1. Protein samples were prepared 72 h post-transfection. Data are reported as the means±SD from three experiments. Statistical significance was assessed by paired *t*-tests between cells transfected with pCMVMycPod-1 or empty vector (P=0.33).

The effect of POD-1-mediated inhibition of *Sf-1/*SF-1 on the steroidogenic function of adrenocortical cells was next assessed by qRT-PCR and immunoblotting analysis of *Star*/StAR mRNA and protein levels, respectively, after transfection of pCMVMycPod-1 (Figures 6 and 7). In F/R cells, *Star*/StAR mRNA and protein levels were significantly reduced by 0.41- (P=0.0021) and 0.19-fold (P=0.0128), respectively, following the transfection of pCMVMycPod-1 compared with transfected controls (Figures 6 and 7, respectively). This correlated with a decrease of SF-1-mediated protein expression in these cells. Although SF-1 expression did not decrease significantly in G cells after POD-1 overexpression, *Star* mRNA levels were significantly reduced in these conditions (P=0.0472; Figure 6). However, StAR protein levels in these cells were not significantly altered by POD-1 overexpression (P=0.40; Figure 7). In agreement with the observations of StAR protein levels, CYP11B2 protein levels in G cells were not significantly modulated by POD-1 overexpression (P=0.33; Figure 8).

## Discussion

SF-1 is a key modulator of adrenal and gonadal steroidogenesis and is known to be regulated by POD-1 in adrenal and gonadal development (23-25). Previously, we showed that POD-1 decreases SF-1 expression in human adrenocortical tumor cells by binding directly to the *SF-1* promoter, thereby inhibiting the activity of both SF-1 and its target *StAR*. Here, we showed that SF-1 was regulated by POD-1 in normal primary adrenocortical cells. Enriched preparations of outer zone glomerulosa cells and of inner zone fasciculata and reticularis cells were readily collected from rat adrenal glands, enabling us to distinguish the individual responses triggered by POD-1 overexpression in these primary cell populations.

Major functional differences exist between the glomerulosa and the fasciculata zones of the rat adrenal cortex (26-28). Whereas the glomerulosa is the site of cell proliferation, maintenance, and differentiation, the fasciculata is the main zone for hormone production (29). Nevertheless, similar levels of SF-1 expression are found in the three zones of the adrenal cortex (30). However, populations of G cells in culture might be composed of different cell types (capsule, subcapsular, and glomerulosa cells), so the difference in the regulation of *Sf-1*and other genes in POD-1-transfected G cells compared with POD-1-transfected F/R cells shown here is not surprising. This difference in regulation can be observed in POD-1-transfected G cells, where *Star*mRNA but not protein expression was down-regulated.

Until recently, the molecular mechanisms underlying functional zonation remained largely unclear. In fact, the formation of the adrenocortical zones in the rat adrenal cortex has been controversial (31). The most prevailing hypothesis is the cell migration theory based on the behavior of incorporated BrdU or tritiated thymidine (32,33). Data support the hypothesis that the adrenal cortex is maintained through the proliferation and clonal replenishment of peripheral cells that undergo centripetal displacement and differentiation in response to endocrine stimulation (23). Nishimoto and collaborators (34,35) showed a clear indication of transcriptome differences between the two cortical zones by microarray analysis. Other findings suggest a relationship between the zonation of the adrenal cortex and the centripetal blood flow, which would generate a gradient of glucocorticoids and different morpho-functional conditions and cell types throughout the adrenal cortex (36).

In the adrenal gland, POD-1 is expressed in the developing capsule of the mouse cortex, which is consistent with its expression in mesenchymal cells during adrenal and gonadal development (13). In the adrenal glands of heterozygous *Pod-1*/LacZ knock-in mice, LacZ expression occurs exclusively in the capsule (23). Moreover, comparative immunohistochemical analysis of heterozygous and homozygous *Pod-1* knockout adrenals revealed that SF-1-positive cells were present in the capsule of homozygous *Pod-1* knockout adrenals. Additionally, POD-1 and GLI1 transcription factors expressed in capsule cells have been suggested to ‘mark' adrenal progenitor cells (37,38). Thus, our results showing that the activity of SF-1 is inhibited in the presence of POD-1 reinforce the notion that POD-1 is a marker of less differentiated cells. Furthermore, cell lineage tracing analyses identified a long-living progenitor population localized along the adrenal cortex, which expresses WT1, GLI1, and POD-1, and is capable of generating steroidogenic cells *in vivo* (24). By contrast, recent data from Wood and co-workers (39) indicate that after the capsule has formed, capsular POD-1-positive cells (but not SF-1-positive cells) give rise to stromal adrenocortical cells but not to steroidogenic cells. However, in the adrenal gland, the role of stromal cells and the relationship between the different cell types in the adrenal cortex have not been investigated. Interestingly, during kidney development, the glomerulogenesis defect observed in null *Pod-1*mice is rescued by the presence of wild-type stromal cells alone, revealing that POD-1 expression in stromal cells is required for the formation of normal nephric structures (40).

We recently analyzed *POD-1* expression in adrenocortical tissue samples, which indicated that this gene is expressed approximately two-fold less in adenomas than in normal tissue and four-fold less in carcinomas than in normal tissue (16). We also found that the expression of *SF-1* was negatively correlated with that of *POD-1* in carcinomas. Moreover, the most significantly enriched pathways for genes that are negatively correlated with *POD-1* in carcinomas from the Kyoto Encyclopedia of Genes and Genomes were those associated with cell cycle genes.

Taken together, our results support the hypothesis that POD-1 inhibits SF-1 expression in adrenal cells, which is consistent with the role of POD-1 as a potential negative regulator not only of adrenocortical tumorigenesis, but also of normal steroidogenesis.
